# Supplementary material for: Genomic signal processing for DNA sequence clustering
Source: PeerJ. 2018 Jan 24;6:e4264. doi: 10.7717/peerj.4264 (PMC5786891; doi:10.7717/peerj.4264)
Supplement: Supplemental Information 1 — Five datasets were used to test the methodology in this paper. These datasets went beyond single gene classification, as discussed in the main body of the article, but also included few very large DNA fragments including whole mitochondrial and bacterial genomes and various medium-and-small-sized viral sequences. A priori groups and k-means clustering results are discussed. [file peerj-06-4264-s002.pdf]

Supplementary file of Mendizabal-Ruiz, et al. *Genetic Signal Processing for DNA Sequence Clustering* (2017).

To test the methodology presented in our paper, we performed various sequence clusterings using different datasets previously tested in the literature (Hoang et al., 2015). These include five genome datasets, i.e. mammal mitochondrion, influenza A virus, human rhinovirus, coronavirus and bacteria. Clustering results are shown below.

In the case of 31 mammal mitochondrial genomes (dataset A), a K7 clustering resulted in the classification of mammal families C1 *Ursidae*, C2 *Bovidae* and *Cetacean*, C3 *Cercopithecidae*, C4 *Hominidae*, C6 *Canidae* and C7 *Felidae*. Cluster 5 contains the *Rodentia* and *Lagomorpha* with the *Erinaceidae* outgroup. This is the result of the early divergence between carnivores, which breaks them in the three tight families, leaving one cluster for the three furthest groups.

The second dataset (B) evaluates the neuraminidase gene, encoded in the sixth segment of the influenza A genome, that is one of two surface genes that identifies the serotypes responsible for virulence and pathogenicity. The K5 clustering resulted in a single swap between a H5N1 and a H1N1 strain in C3 and C4 respectively. This result is similar to that of *k*-mer clustering method tested by Hoang, et al.

The Human rhinovirus dataset (C) did not cluster according to the four genome groups analyzed in HRV-A, HRV-B, HRV-C and HEV-C as outgroup. Instead, K4 clustering divided HRV-A in three groups, with C1 sharing both furthest branches of HRV-A, HRV-C and HEV-C, and C3 with all of HRV-B (Palmenberg et al., 2009). The great diversity of HRV-A created compact clusters distant enough from each other to cluster the loosest groups in each other.

Coronavirus complete genome dataset (D) resulted in accurate K5 clustering with the sole exception of SARS strain ZJ01. Most viruses clustered according to the species they affect. Murine hepatitis virus and Bovine coronavirus, two groups typically grouped together, clustered separately in C2 and C3 respectively, and C1 clustered most Human coronavirus.

Finally, we analyzed eight families of whole bacterial genomes (dataset E). The most interesting result is the segregation of both *E. coli* strains into the separate C5 and C7 clusters, and apart from the *Shigellas* and *Yersinias*, that clustered in C8. Both *Bacilli* genera also clustered separately in C1 and C6. The three remaining clusters held two bacterial families, according to their phylogenetic relatedness.

Dataset A<br>Mammals					
Cluster	Number	*A priori*<br>Group	NCBI<br>Accession No.	Name	Length
C1	23	6	DQ402478	Black bear	16832
C1	24	6	AF303110	Brown bear	16868
C1	25	6	AF303111	Polar bear	17020
C1	26	6	EF212882	Giant panda	17017
C2	13	2	AJ002189	Pig	16727
C2	14	2	AF010406	Sheep	16680
C2	15	2	AF533441	Goat	16616
C2	16	2	V00654	Cow	16640
C2	17	2	AY488491	Buffalo	16338
C2	31	2	X72204	Blue whale	16402
C2	21	3	X97336	Indian rhinoceros	16964
C2	22	3	Y07726	White rhinoceros	16829
C3	4	1	X99256	Gibbon	16521
C3	5	1	Y18001	Baboon	16389
C3	6	1	AY863426	Vervet monkey	16586
C3	7	1	NC 002764	Macaca thibetana	17447
C3	8	1	D38115	Bornean orangutan	16389
C3	9	1	NC 002083	Sumatran orangutan	16499
C4	1	1	V00662	Human	16569
C4	2	1	D38116	Pigmy chimpanzee	16563
C4	3	1	D38113	Common chimpanzee	16554
C4	10	1	D38114	Gorilla	16472
C5	27	5	AJ001588	Rabbit	16805
C5	28	7	X88898	Hedgehog	17245
C5	29	4	AJ001562	Dormouse	16507
C5	30	4	AJ238588	Squirrel	16602
C6	12	6	U96639	Dog	17009
C6	18	6	EU442884	Wolf	16355
C7	11	6	U20753	Cat	16364
C7	19	6	EF551003	Tiger	16774
C7	20	6	EF551002	Leopard	16990

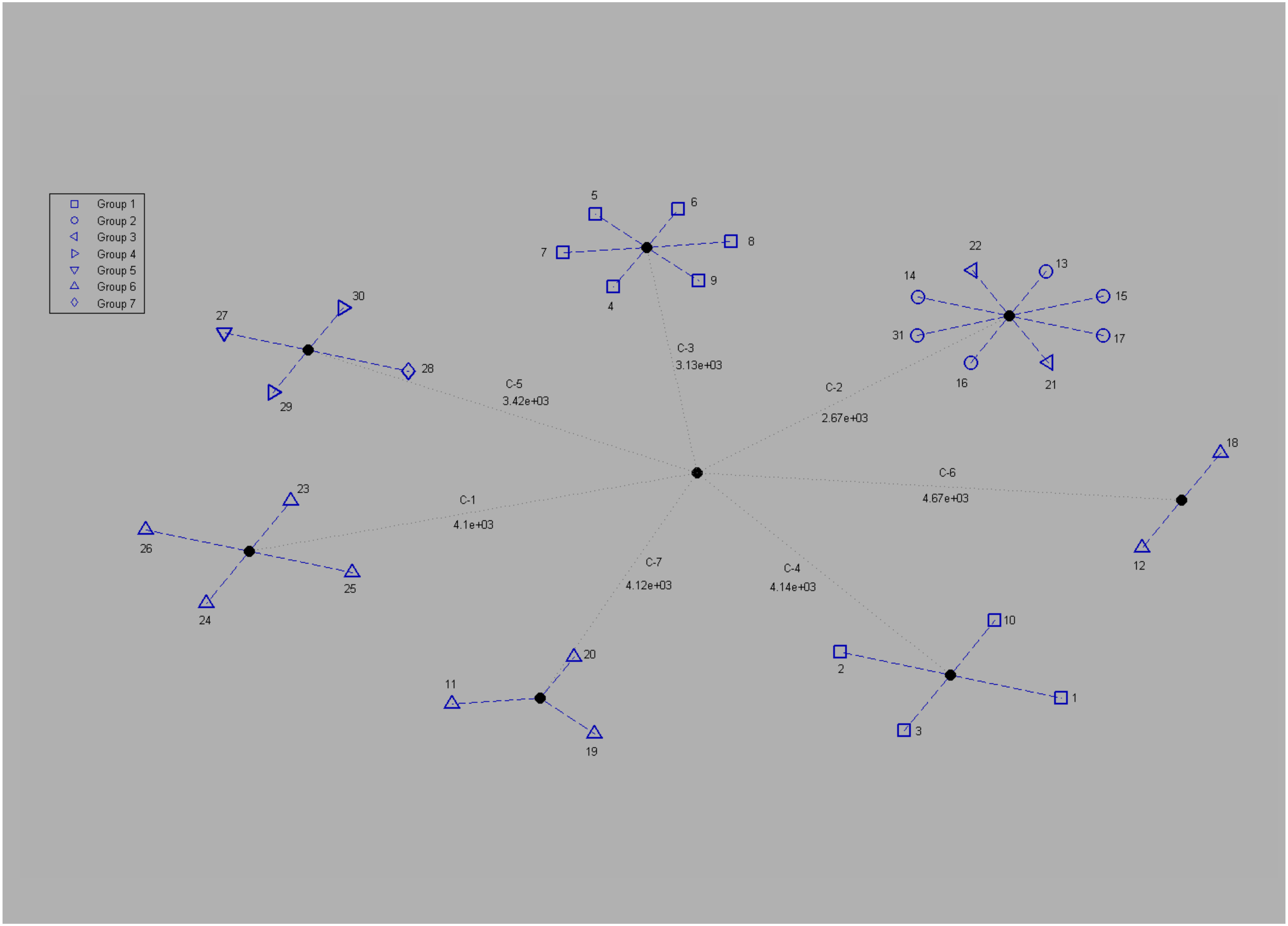

Group 1
Group 2
Group 3
Group 4
Group 5
Group 6
Group 7
C-1
4.1e+03
C-2
2.67e+03
C-3
3.13e+03
C-4
4.14e+03
C-5
3.42e+03
C-6
4.67e+03
C-7
4.12e+03

Dataset B<br>Influenza A viruses					
Cluster	Number	*A priori* Group	NCBI Accession No.	Name	Length
C1	25	3	JX081142	A/emperor goose/Alaska/44297-260/2007(H2N2)	1457
C1	26	3	DQ017487	A/mallard/Postdam/178-4/1983(H2N2)	1467
C1	27	3	CY005540	A/duck/Hong Kong/319/1978(H2N2)	1467
C2	28	4	KF259734	A/chicken/Rizhao/713/2013(H7N9)	1398
C2	29	4	KF938945	A/chicken/Jiangsu/1021/2013(H7N9)	1404
C2	30	4	KF259688	A/duck/Jiangxi/3096/2009(H7N9)	1413
C2	31	4	KC609801	A/wild duck/Korea/SH19-47/2010(H7N9)	1426
C2	32	4	CY186004	A/mallard/Minnesota/AI09-3770/2009(H7N9)	1422
C2	33	4	CY014788	A/turkey/Minnesota/1/1988(H7N9)	1460
C3	12	2	CY149630	A/thick-billed murre/Canada/1871/2011(H1N1)	1433
C3	13	2	CY140047	A/mallard/Minnesota/Sg-00620/2008(H1N1)	1433
C3	14	2	EU026046	A/mallard/Maryland/352/2002(H1N1)	1433
C3	15	2	FJ357114	A/mallard/Maryland/26/2003(H1N1)	1433
C3	16	2	CY138562	A/mallard/Nova Scotia/00088/2010(H1N1)	1422
C3	17	2	HM370969	A/turkey/Ontario/FAV110-4/2009(H1N1)	1419
C3	18	2	KM244078	A/turkey/Virginia/4135/2014(H1N1)	1410
C3	20	2	AB470663	A/duck/Hokkaido/w73/2007(H1N1)	1422
C3	21	2	HQ897966	A/mallard/Korea/KNU YP09/2009(H1N1)	1410
C3	22	2	GQ411894	A/dunlin/Alaska/44421-660/2008(H1N1)	1413
C3	23	2	AM157358	A/mallard/France/691/2002(H1N1)	1413
C3	24	2	AB546159	A/pintail/Miyagi/1472/2008(H1N1)	1410
C3	2	1	GU186511	A/turkey/VA/505477-18/2007(H5N1)	1370
C4	19	2	KC608160	A/duck/Guangxi/030D/2009(H1N1)	1398
C4	1	1	FM177121	A/chicken/Germany/R3234/2007(H5N1)	1370
C4	3	1	AF509102	A/Chicken/Hong Kong/822.1/01 (H5N1)	1366
C4	4	1	HQ185381	A/chicken/Eastern China/XH222/2008(H5N1)	1350
C4	5	1	HQ185383	A/duck/Eastern China/JS017/2009(H5N1)	1350
C4	6	1	AB684161	A/chicken/Miyazaki/10/2011(H5N1)	1350
C4	7	1	JF699677	A/mandarin duck/Korea/K10-483/2010(H5N1)	1350
C4	8	1	AM914017	A/domestic duck/Germany/R1772/2007(H5N1)	1350
C4	9	1	KF572435	A/wild bird/Hong Kong/07035-1/2011(H5N1)	1350
C4	10	1	EU635875	A/chicken/Yunnan/chuxiong01/2005(H5N1)	1350
C4	11	1	EF541464	A/chicken/Korea/es/2003(H5N1)	1350
C5	34	5	EU500854	A/American black duck/NB/2538/2007(H7N3)	1453
C5	35	5	AY646080	A/chicken/British Columbia/GSC human B/04(H7N3)	1453
C5	36	5	CY076231	A/American green-winged teal/California/44242-906/2007(H7N3)	1420
C5	37	5	CY129336	A/American black duck/New Brunswick/02490/2007(H7N3)	1428
C5	38	5	CY039321	A/avian/Delaware Bay/226/2006(H7N3)	1434

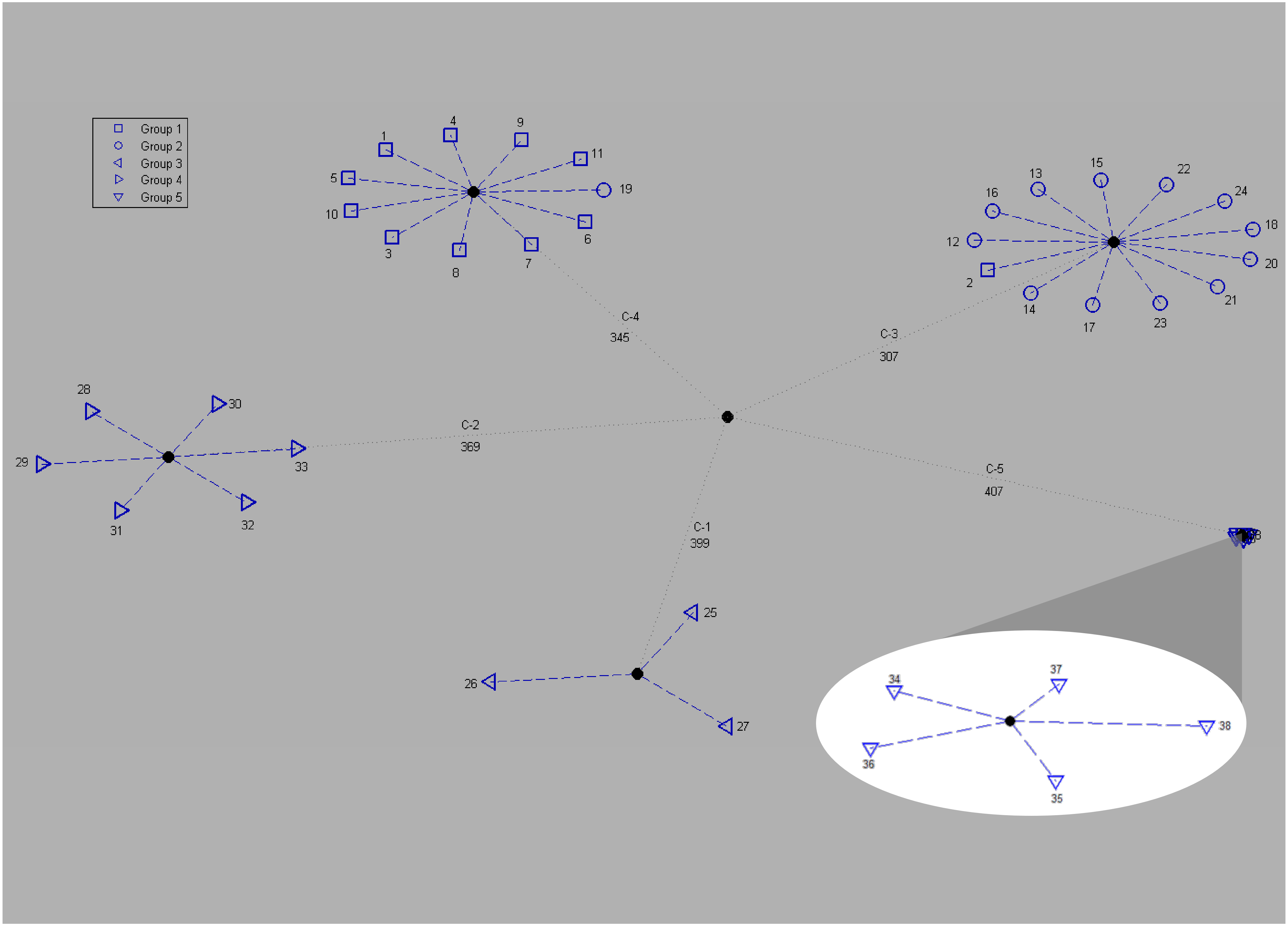

Group 1
Group 2
Group 3
Group 4
Group 5
C-4
345
C-3
307
C-2
369
C-5
407
C-1
399
1
2
3
4
5
6
7
8
9
10
11
12
13
14
15
16
17
18
19
20
21
22
23
24
25
26
27
28
29
30
31
32
33
34
35
36
37
38

Dataset C<br>Human rhinovirus					
Cluster	Number	*A priori* Group	NCBI Accession No.	Name	Length
C1	1	4	AF499637	HEV_cva-13*	7458
C1	2	4	AF546702	HEV_cva-21*	7406
C1	115	4	V01149	HEV_pv-1m*	7440
C1	24	3	EF077279	C_nat001*	6944
C1	25	3	EF077280	C_nat045*	7015
C1	31	3	EF186077	C_qpm*	7134
C1	32	3	EF582385	C_c024*	7099
C1	33	3	EF582386	C_c025*	7114
C1	34	3	EF582387	C_c026*	7086
C1	27	1	EF173415	A_hrv-12*	7124
C1	37	1	FJ445113	A_hrv-08	7108
C1	55	1	FJ445132	A_hrv-45	7114
C1	59	1	FJ445136	A_hrv-51	7152
C1	65	1	FJ445142	A_hrv-58	7140
C1	70	1	FJ445147	A_hrv-65	7162
C1	74	1	FJ445152	A_hrv-71	7161
C1	78	1	FJ445156	A_hrv-80	7138
C1	87	1	FJ445165	A_hrv-89-f09	7150
C1	88	1	FJ445166	A_hrv-89-f08	7152
C1	92	1	FJ445170	A_hrv-95	7110
C1	98	1	FJ445176	A_hrv-07	7146
C1	106	1	FJ445184	A_hrv-89	7152
C1	17	1	DQ473504	A_hrv-88*	7143
C1	18	1	DQ473505	A_hrv-36*	7141
C1	19	1	DQ473506	A_hrv-46*	7149
C1	20	1	DQ473507	A_hrv-53*	7143
C1	21	1	DQ473508	A_hrv-28*	7148
C2	9	1	DQ473491	A_hrv-41*	7145
C2	10	1	DQ473492	A_hrv-73*	7140
C2	11	1	DQ473493	A_hrv-15*	7134
C2	22	1	DQ473510	A_hrv-75*	7137
C2	38	1	FJ445114	A_hrv-09-f01	7134
C2	39	1	FJ445115	A_hrv-09-f02	7133
C2	40	1	FJ445116	A_hrv-13	7140
C2	41	1	FJ445117	A_hrv-13-f03	7143
C2	43	1	FJ445119	A_hrv-19	7135
C2	45	1	FJ445122	A_hrv-22	7129
C2	50	1	FJ445127	A_hrv-32	7133

C2	54	1	FJ445131	A_hrv-43	7129
C2	66	1	FJ445143	A_hrv-60	7139
C2	67	1	FJ445144	A_hrv-61	7139
C2	72	1	FJ445149	A_hrv-67	7135
C2	82	1	FJ445160	A_hrv-82	7123
C2	93	1	FJ445171	A_hrv-96	7134
C2	99	1	FJ445177	A_hrv-09	7132
C2	102	1	FJ445180	A_hrv-38	7136
C2	103	1	FJ445181	A_hrv-64	7129
C2	107	1	FJ445185	A_hrv-94	7132
C3	4	2	DQ473485	B_hrv-03*	7208
C3	5	2	DQ473486	B_hrv-06*	7216
C3	6	2	DQ473488	B_hrv-48*	7214
C3	7	2	DQ473489	B_hrv-70*	7223
C3	8	2	DQ473490	B_hrv-04*	7212
C3	28	2	EF173420	B_hrv-17*	7219
C3	29	2	EF173423	B_hrv-37*	7216
C3	30	2	EF173425	B_hrv-93*	7215
C3	36	2	FJ445112	B_hrv-05	7212
C3	47	2	FJ445124	B_hrv-26	7211
C3	53	2	FJ445130	B_hrv-42	7223
C3	60	2	FJ445137	B_hrv-52-f10	7216
C3	73	2	FJ445151	B_hrv-69	7211
C3	75	2	FJ445153	B_hrv-72	7216
C3	77	2	FJ445155	B_hrv-79	7224
C3	83	2	FJ445161	B_hrv-83	7230
C3	84	2	FJ445162	B_hrv-84	7201
C3	86	2	FJ445164	B_hrv-86	7213
C3	90	2	FJ445168	B_hrv-91	7221
C3	91	2	FJ445169	B_hrv-92	7233
C3	94	2	FJ445172	B_hrv-97	7207
C3	96	2	FJ445174	B_hrv-99	7208
C3	108	2	FJ445186	B_hrv-27	7217
C3	109	2	FJ445187	B_hrv-35	7224
C3	110	2	FJ445188	B_hrv-52	7216
C3	113	2	L05355	B_hrv-14*	7212
C4	3	1	AY751783	A_hrv-39*	7137
C4	12	1	DQ473494	A_hrv-74*	7120
C4	13	1	DQ473496	A_hrv-49*	7106
C4	14	1	DQ473497	A_hrv-23*	7025
C4	15	1	DQ473499	A_hrv-44*	7123
C4	16	1	DQ473500	A_hrv-59*	7135
C4	23	1	DQ473511	A_hrv-55*	7036

C4	26	1	EF173414	A_hrv-11*	7125
C4	35	1	FJ445111	A_hrv-01	7137
C4	42	1	FJ445118	A_hrv-18	7119
C4	44	1	FJ445121	A_hrv-21	7134
C4	46	1	FJ445123	A_hrv-25	7126
C4	48	1	FJ445125	A_hrv-29	7123
C4	49	1	FJ445126	A_hrv-31	7131
C4	51	1	FJ445128	A_hrv-33	7133
C4	52	1	FJ445129	A_hrv-40	7138
C4	56	1	FJ445133	A_hrv-47	7132
C4	57	1	FJ445134	A_hrv-49-f04	7109
C4	58	1	FJ445135	A_hrv-50	7118
C4	61	1	FJ445138	A_hrv-54	7134
C4	62	1	FJ445139	A_hrv-54-f05	7133
C4	63	1	FJ445140	A_hrv-56	7136
C4	64	1	FJ445141	A_hrv-57	7134
C4	68	1	FJ445145	A_hrv-62	7131
C4	69	1	FJ445146	A_hrv-63	7141
C4	71	1	FJ445148	A_hrv-66	7139
C4	76	1	FJ445154	A_hrv-77	7136
C4	79	1	FJ445157	A_hrv-81	7116
C4	80	1	FJ445158	A_hrv-81-f06	7116
C4	81	1	FJ445159	A_hrv-81-f07	7116
C4	85	1	FJ445163	A_hrv-85	7140
C4	89	1	FJ445167	A_hrv-90	7124
C4	95	1	FJ445173	A_hrv-98	7133
C4	97	1	FJ445175	A_hrv-100	7140
C4	100	1	FJ445178	A_hrv-10	7137
C4	101	1	FJ445179	A_hrv-30	7093
C4	104	1	FJ445182	A_hrv-76	7128
C4	105	1	FJ445183	A_hrv-78	7145
C4	111	1	FJ445189	A_hrv-34	7119
C4	112	1	FJ445190	A_hrv-24	7132
C4	114	1	L24917	A_hrv-16*	7124
C4	116	1	X02316	A_hrv-02*	7102

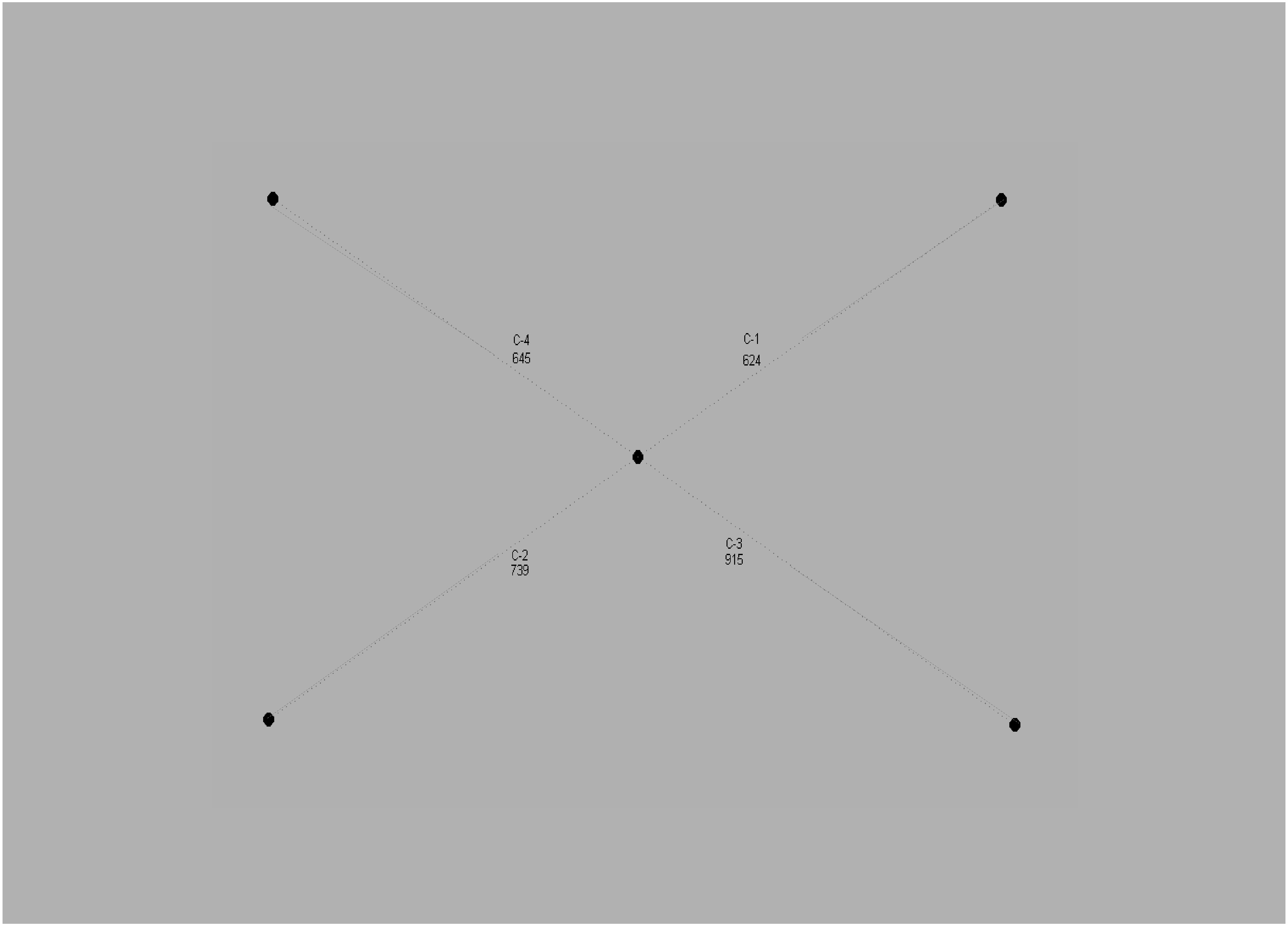
C-4
645
C-1
624
C-2
739
C-3
915

Dataset D<br>Coronavirus					
Cluster	No.	*A priori* Group	NCBI Accession No.	Name	Length
C1	16	1	AF304460	Human coronavirus 229E	27317
C1	17	1	AF353511	Porcine epidemic diarrhea virus strain	28033
C1	29	1	NC_005831	Human coronavirus NL63	27553
C1	30	5	NC_006577	Human coronavirus HKU1	29926
C1	13	4	AY297028	SARS coronavirus ZJ01	29715
C2	20	2	AF208067	Murine hepatitis virus strain ML-10	31233
C2	21	2	NC_001846	Murine hepatitis virus	31357
C2	22	2	AF201929	Murine hepatitis virus strain 2	31276
C2	23	2	AF208066	Murine hepatitis virus strain Penn 97-1	31112
C3	24	2	AY391777	Human coronavirus OC43	30738
C3	25	2	U00735	Bovine coronavirus strain Mebus	31032
C3	26	2	AF220295	Bovine coronavirus strain Quebec	31100
C3	27	2	AF391542	Bovine coronavirus isolate BCoV-LUN	31028
C3	28	2	NC_003045	Bovine coronavirus	31028
C4	18	3	NC_001451	Avian infectious bronchitis virus	27608
C4	19	3	EU095850	Turkey coronavirus isolate MG10	27657
C5	31	6	NC_001564	Cell fusing agent virus	10695
C5	32	6	NC_001512	O'nyong-nyong virus	11835
C5	33	6	NC_001544	Ross River virus	11657
C5	34	6	NC_004102	Hepatitis C virus	9646
C6	1	4	AY283794	SARS coronavirus isolate SIN2500	29711
C6	2	4	AY283797	SARS coronavirus isolate SIN2748	29706
C6	3	4	AY283798	SARS coronavirus isolate SIN2774	29711
C6	4	4	AY283796	SARS coronavirus isolate SIN2679	29711
C6	5	4	AY291451	SARS coronavirus TW1	29729
C6	6	4	AY283795	SARS coronavirus isolate SIN2677	29705
C6	7	4	AY278741	SARS coronavirus Urbani	29727
C6	8	4	AY278488	SARS coronavirus BJ01	29725
C6	9	4	AY278491	SARS coronavirus HKU-39849	29742
C6	10	4	AY278554	SARS coronavirus CUHK-W1	29736
C6	11	4	AY282752	SARS coronavirus CUHK-Su10	29736
C6	12	4	NC_004718	SARS coronavirus TOR2	29751
C6	14	4	AY572034	SARS coronavirus civet007	29540
C6	15	4	AY572035	SARS coronavirus civet010	29518

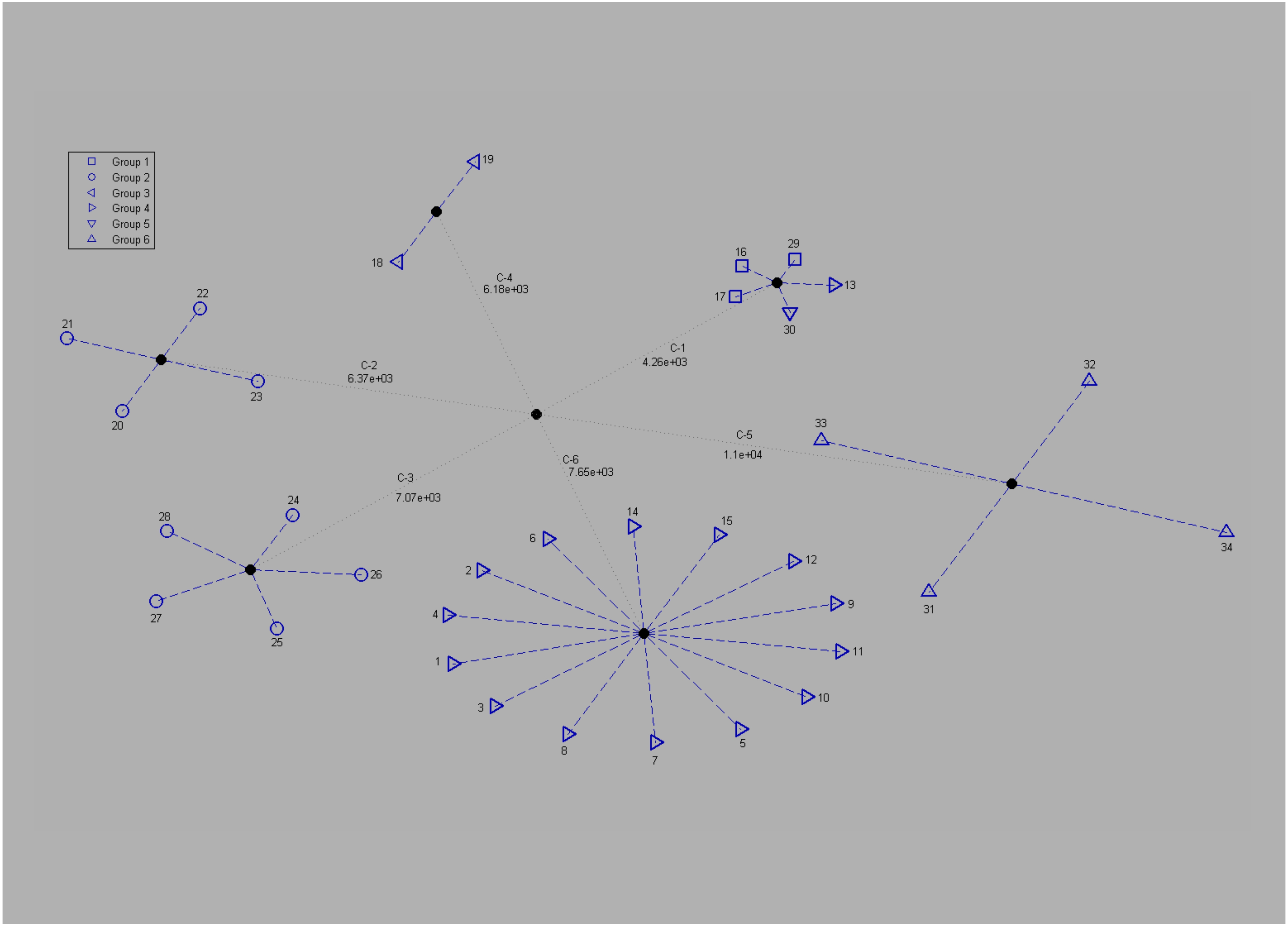

Group 1
Group 2
Group 3
Group 4
Group 5
Group 6
C-1
4.26e+03
C-2
6.37e+03
C-3
7.07e+03
C-4
6.18e+03
C-5
1.1e+04
C-6
7.65e+03
1
2
3
4
5
6
7
8
9
10
11
12
13
14
15
16
17
18
19
20
21
22
23
24
25
26
27
28
29
30
31
32
33
34

Dataset E Bacteria					
Cluster	Name	*A priori* Group	NCBI Accession No.	Name	Length
C1	21	7	AE017225.1	*Bacillus anthracis Sterne*	5228663
C1	22	7	CP001215.1	*Bacillus anthracis CDC 684*	5230115
C2	1	1	AM295250.1	*Staphylococcus carnosus carnosus*	2566424
C2	2	1	AE015929.1	*Staphylococcus epidermidis ATCC 12228*	2499279
C2	3	1	AP006716.1	*Staphylococcus haemolyticus JCSC1435*	2685015
C2	4	1	CP001837.1	*Staphylococcus lugdunensis HKU09-01*	2658366
C2	5	2	CP000246.1	*Clostridium perfringens ATCC 13124*	3256683
C2	6	2	CP000312.1	*Clostridium perfringens SM101*	2897393
C2	7	2	BA000016.3	*Clostridium perfringens 13*	3031430
C3	13	5	CP001151.1	*Rhodobacter sphaeroides KD131*	1287647
C3	14	5	CP000578.1	*Rhodobacter sphaeroides ATCC 17029*	1219053
C3	15	6	CP000048.1	*Borrelia hermsii DAH*	922307
C3	16	6	CP000049.1	*Borrelia duttonii Ly*	931674
C3	17	6	CP000976.1	*Borrelia turicatae 91E135*	917330
C3	18	6	CP000993.1	*Borrelia recurrentis A1*	930981
C4	8	3	AM260480.1	*Ralstonia eutropha H16*	2912490
C4	9	3	CP000091.1	*Ralstonia eutropha JMP134*	2726152
C4	10	4	CP000527.1	*Desulfovibrio vulgaris DP4*	3462887
C4	11	4	CP002297.1	*Desulfovibrio vulgaris RCH1*	3532052
C4	12	4	AE017285.1	*Desulfovibrio vulgaris vulgaris Hildenborough*	3570858
C5	23	8	CP001671.1	*Escherichia coli ABU 83972*	5131397
C6	19	7	CP001598.1	*Bacillus anthracis A0248*	5227419
C6	20	7	AE016879.1	*Bacillus anthracis Ames*	5227293
C7	24	8	CP000468.1	*Escherichia coli APEC O1*	5082025
C8	25	8	AE005674.1	*Shigella flexneri 2a 301*	4607203
C8	26	8	CP001383.1	*Shigella flexneri 2002017*	4650856
C8	27	8	CP001593.1	*Yersinia pestis Z176003*	4553586
C8	28	8	AE009952.1	*Yersinia pestis KIM10+*	4600755
C8	29	8	AL590842.1	*Yersinia pestis CO92*	4653728
C8	30	8	CP001585.1	*Yersinia pestis D106004*	4640720

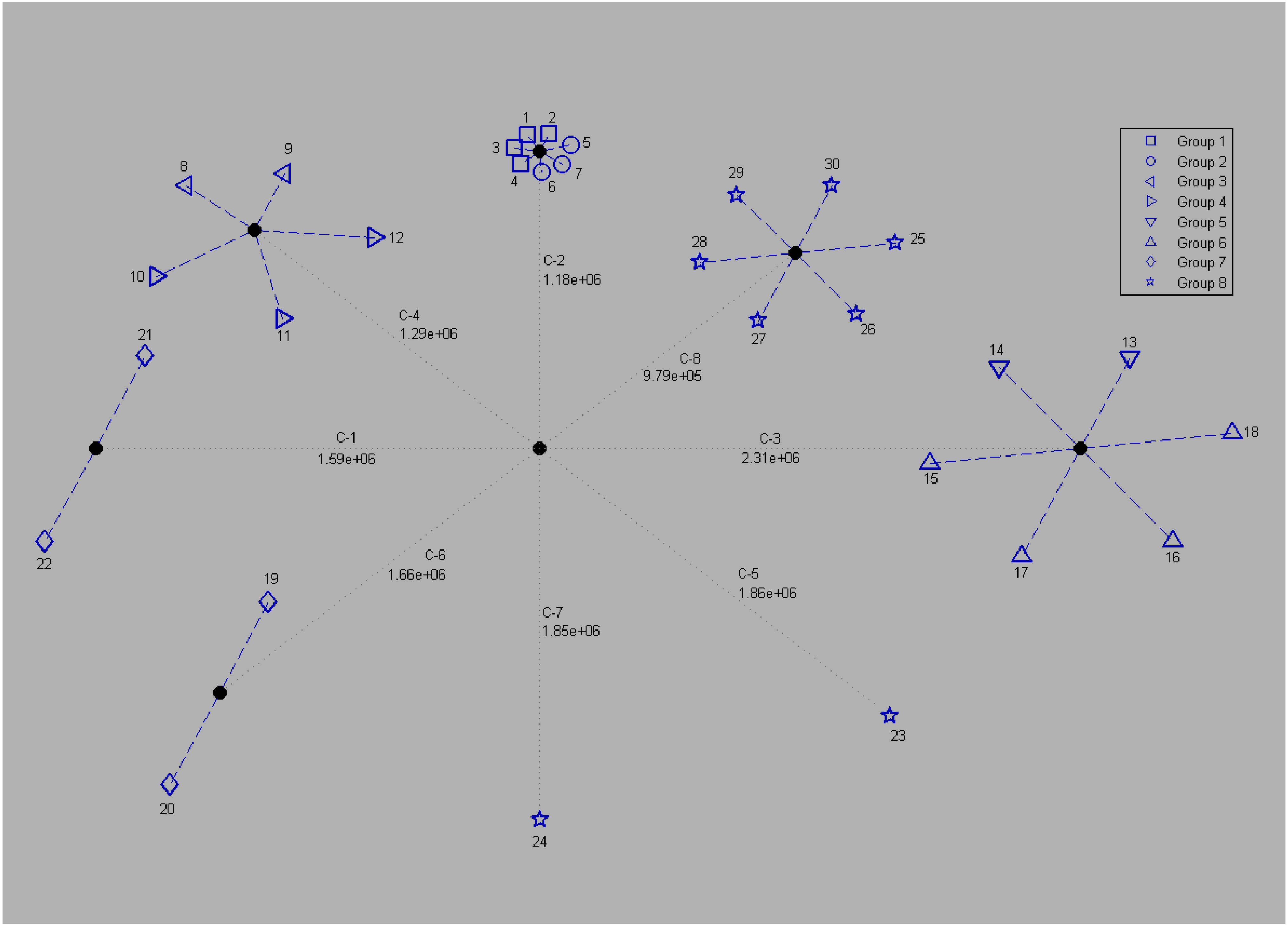
Group 1
Group 2
Group 3
Group 4
Group 5
Group 6
Group 7
Group 8
C-1
1.59e+06
C-2
1.18e+06
C-3
2.31e+06
C-4
1.29e+06
C-5
1.86e+06
C-6
1.66e+06
C-7
1.85e+06
C-8
9.79e+05
1
2
3
4
5
6
7
8
9
10
11
12
13
14
15
16
17
18
19
20
21
22
23
24
25
26
27
28
29
30

References:

Hoang, T., Yin, C., Zheng, H., Yu, C., Lucy He, R., & Yau, S. S.-T. (2015). A new method to cluster DNA sequences using Fourier power spectrum. *Journal of Theoretical Biology*, *372*, 135–145. https://doi.org/10.1016/j.jtbi.2015.02.026

Palmenberg, A. C., Spiro, D., Kuzmickas, R., Wang, S., Djikeng, A., Rathe, J. A., … Liggett, S. B. (2009). Sequencing and Analyses of All Known Human Rhinovirus Genomes Reveal Structure and Evolution. *Science*, *324*(5923), 55–59. https://doi.org/10.1126/science.1165557
